# Association between cytology and endoscopy of guttural pouches: a prospective study on 173 horses

**DOI:** 10.1093/jvimsj/aalag179

**Published:** 2026-08-26

**Authors:** Pauline Barbazanges, Eric A Richard, Charlotte Debonnaire, Gabin Le Digarcher, Louise C Lemonnier, Marie-Pierre Toquet, Anne Couroucé

**Affiliations:** Equine Internal Medicine, PB EQUIMED, Mérignac, France; Department of Clinical Sciences, Equine Internal Medicine, Oniris VetAgroBio Nantes, Nantes, France; Equine Health, LABÉO (Frank Duncombe), Caen, France; BIOTARGEN UR7450, Normandie Équine Vallée, GIS Centaure, Université de Caen Normandie, Normandie Université, Caen, France; Department of Clinical Sciences, Equine Internal Medicine, Oniris VetAgroBio Nantes, Nantes, France; Department of Clinical Sciences, Equine Internal Medicine, Oniris VetAgroBio Nantes, Nantes, France; Equine Internal Medicine, MBVet – Centre Vétérinaire Hospitalier Equin de Méheudin, Ecouché-les-Vallées, France; Equine Health, LABÉO (Frank Duncombe), Caen, France; BIOTARGEN UR7450, Normandie Équine Vallée, GIS Centaure, Université de Caen Normandie, Normandie Université, Caen, France; Department of Clinical Sciences, Equine Internal Medicine, Oniris VetAgroBio Nantes, Nantes, France; Equine Health, LABÉO (Frank Duncombe), Caen, France; BIOTARGEN UR7450, Normandie Équine Vallée, GIS Centaure, Université de Caen Normandie, Normandie Université, Caen, France

**Keywords:** clinical pathology, cytology, endoscopy, equid, respiratory tract

## Abstract

**Background:**

Guttural pouch (GP) disorders are clinically important in horses, yet the relationship between endoscopic findings and GP cytology remains poorly defined.

**Hypothesis/Objectives:**

To establish reference cytological values for GP lavage fluid in horses and to investigate associations between inflammatory cytology and endoscopic findings.

**Animal:**

Asymptomatic racing horses sampled in the field and horses referred for investigation of respiratory disorders.

**Methods:**

Prospective cross-sectional study. Horses underwent clinical examination, upper airway endoscopy, and bilateral transendoscopic GP lavage. A GP inflammation score (0-15) was developed based on endoscopic appearance in the GP. Cytology was performed on GP pooled samples. Control horses were defined by the absence of clinical, hematologic, microbiological, and endoscopic abnormalities. Associations were analyzed using nonparametric tests.

**Results:**

A total of 173 horses were included, of which 19 were controls. Control GP fluid cytology exhibited no or rare cells (median 30 cells/mm^3^; range 10-90) which were either neutrophils, macrophages, or lymphocytes. The remaining 154 horses were classified as “abnormal” (ABN) and subdivided into 2 groups according to GP fluid neutrophil proportions: group ABN1 (<1% neutrophils, *n* = 101, median 30 cells/mm^3^; range 5-360) and group ABN2 (neutrophils ≥ 1%, *n* = 53, median 100 cells/mm^3^; range 30-600). Horses in the ABN2 group showed more frequent GP lymphoid hyperplasia, secretions, and purulent exudate than horses in ABN1.

**Conclusions and clinical importance:**

Cytological inflammation correlates with endoscopic evidence of GP inflammation.

## Introduction

Guttural pouches (GPs)—diverticula of the Eustachian tubes—are unique anatomical structures with relevant clinical implications in equine medicine, particularly in cases of GP empyema secondary to strangles or GP mycosis.^1^ Due to their proximity to critical neurovascular structures, disorders affecting the GP can have important clinical consequences.^2^^,^^3^

Endoscopy remains the primary diagnostic tool for evaluating the GP, providing direct visualization of the mucosa and changes such as empyema, mycosis, or neoplasia.^1^^,^^3^ Inflammation of the GP can manifest as lymphoid hyperplasia, exudate, hyperemia, or lymphadenopathy, all of which are visible on endoscopy. However, endoscopic examination alone may be insufficient to fully characterize the nature or severity of disease, particularly in subclinical or early-stage conditions.

Cytological analysis of GP lavage fluid offers a complementary diagnostic approach, allowing for the assessment of non-macroscopically visible components present within the pouches. Guttural pouch lavage is the method of choice used for obtaining a liquid sample, mainly for microbial investigations. Percutaneous washes of the GP were previously described as useful, minimally invasive, and associated with minimal risk of bacterial or cell contamination that might accompany scope insertion.^4–6^ However, transendoscopic washes of the GP have been used more often in practice, and this technique is part of the routine evaluation of equine upper airway disorders.

Studies have confirmed that leukocytes are relatively sparse in the washings from normal GPs (neutrophils < 5% of total cells^4^^,^^7^^,^^8^), although they are highly prevalent within washings collected from horses with *Streptococcus equi* in their GP.^9^ Moreover, other factors such as head elevation or exercise can affect the cytology of GP.^4^^,^^6^

Despite the routine use of both endoscopy and cytology in clinical practice, there is a lack of systematic studies examining the correlation between the cytological profile of GP samples and their corresponding endoscopic appearance. This study aims to investigate the cytological characteristics of GP samples in a large cohort of horses by determining the cytological reference values of this cohort. The second objective was to look for an association between inflammatory cytology in the GP and endoscopic findings. We hypothesize that abnormal findings on endoscopy (presence of pus, signs of inflammation) will be predictive of an inflammatory cytology.

## Materials and methods

The current study was based on a previous large research project.^10^ The study group included convenience sampling of 90 French Standardbred racehorses in active training between October 2020 and January 2023, and voluntary recruitment of 84 client-owned horses referred to the hospital between February 2021 and May 2023 for decreased performance and/or respiratory disease. Horses with signs of systemic illness or other causes of decreased performance were excluded. Additional data are provided in the Appendix.

Horses were sampled at least 24 h after the last training session or race. Horses were either sampled in their usual environment (at their stables) or at the hospital after brief (<2 h) transportation.

A 0-4 score was assigned for pharyngeal lymphoid hyperplasia (PLH) observed at the level of the nasopharyngeal mucosa and dorsal pharyngeal recess.^11^

A scoring system ranging from 0 to 15 was developed to evaluate GP inflammation, adapted from a previously described score.^12^ Mucosal appearance was assessed for signs of inflammation, including the presence of hyperemia (1 point) or follicular hyperplasia (2 points). The presence and character of exudate were also evaluated: mucoid (1 point) or purulent (2 points) secretions. The quantity of exudate was graded as follows: minimal presence (few spots) received 1 point, exudate coalescing in filamentous secretions received 2 points, and extensive accumulation received 3 points. Additionally, retropharyngeal lymph nodes were examined, and enlargement was scored as 3 points. A single cumulative score was assigned based on the combined evaluation of both right and left GPs.

The aspirated fluid of both pouches was pooled. Mycology and a polymerase chain reaction (PCR) for *S equi* subsp. *equi* were systematically performed on all samples. Bacteriological testing was carried out only on samples selected at the clinician’s request. Detailed methodological information is provided in the Appendix.

Horses were defined as controls based on (1) the absence of evidence of systemic signs of infection (anorexia, lethargy, and fever), respiratory signs, and/or hematologic abnormalities compatible with infection; (2) a negative bacteriology result on tracheal wash (TW); (3) the absence of visible abnormalities on GP endoscopy; and (4) a negative PCR for *S equi* subsp. *equi* on pooled GP samples (CTL group). Horses with ≥ 1 of these criteria were considered as “abnormal” (ABN group).

Statistical analyses were conducted using GraphPad Prism 10.6.1, with data reported as median (IQR), univariable associations assessed by chi-square test with Yates’ correction, group comparisons and correlations performed using Mann–Whitney *U* and Spearman’s rank tests, correlation strength categorized by predefined thresholds, and significance set at *P* < .05.

## Results

### Study group

The cohort included 174 horses and was composed of 95 Standardbred racehorses (54.6%), 26 sport horses (14.9%), and 53 leisure horses (30.5%). Horses were aged from 2 to 25 years (median 6 years), with a total of 41 entire males (23.6%), 67 geldings (38.5%), and 66 females (37.9%). Sampling was performed at their stables for 91 horses and 83 horses were sampled at the hospital. Horse characteristics are described in Table S1.

Among the 174 horses sampled, 1 horse was excluded because samples were uninterpretable on receipt at the laboratory. Overall, 173 horses were included.

### Endoscopic findings

#### Pharynx

Pharyngeal lymphoid hyperplasia was graded in 168 horses. Fifty-nine had grade 0, 28 had grade 1, 26 had grade 2, 21 had grade 3, and 34 had grade 4.

Racehorses had a significantly higher PLH grade (median 2.75; IQ1 1, IQ3 4; min 0, max 4) compared to non-racing horses (median 0; IQ1 0, IQ3 1; min 0, max 4; *P* < .001; Figure S1).

A moderate-to-strong negative correlation was observed between age and PLH grade (Spearman’s ρ = −0.642; 95% CI, −0.725 to −0.540; *P* < .001). No difference was observed in PLH grade between horses living in pasture and horses in box.

#### Guttural pouches

**Figure 1:**
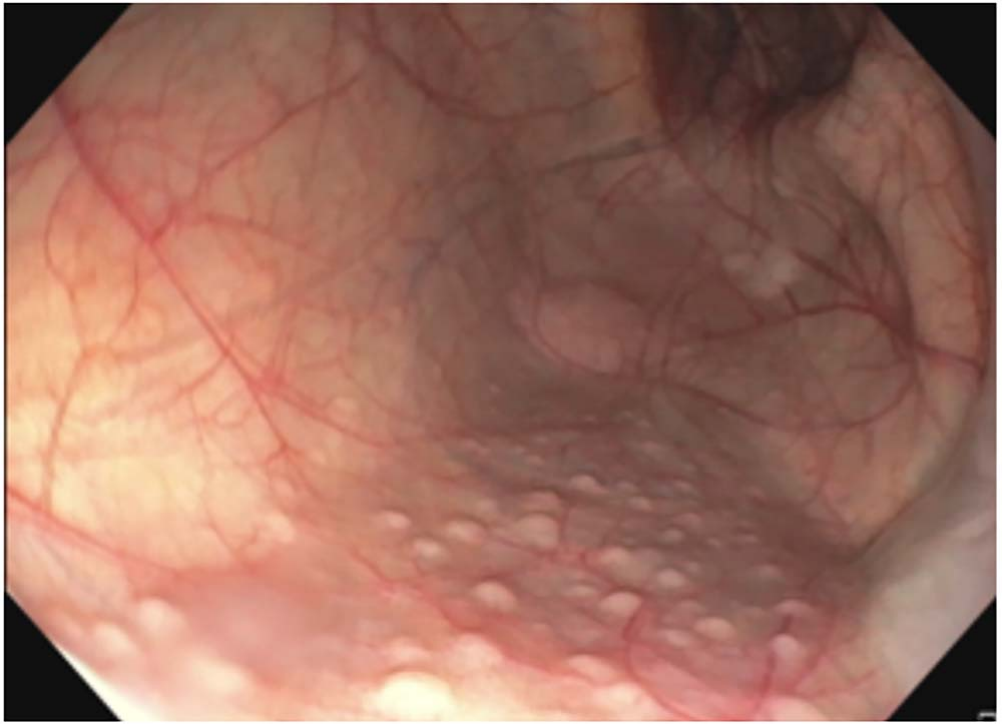
Lymphoid hyperplasia on the floor of the medial compartment of the right GP (GP inflammation score 2). Abbreviation: GP = guttural pouch.

**Figure 2:**
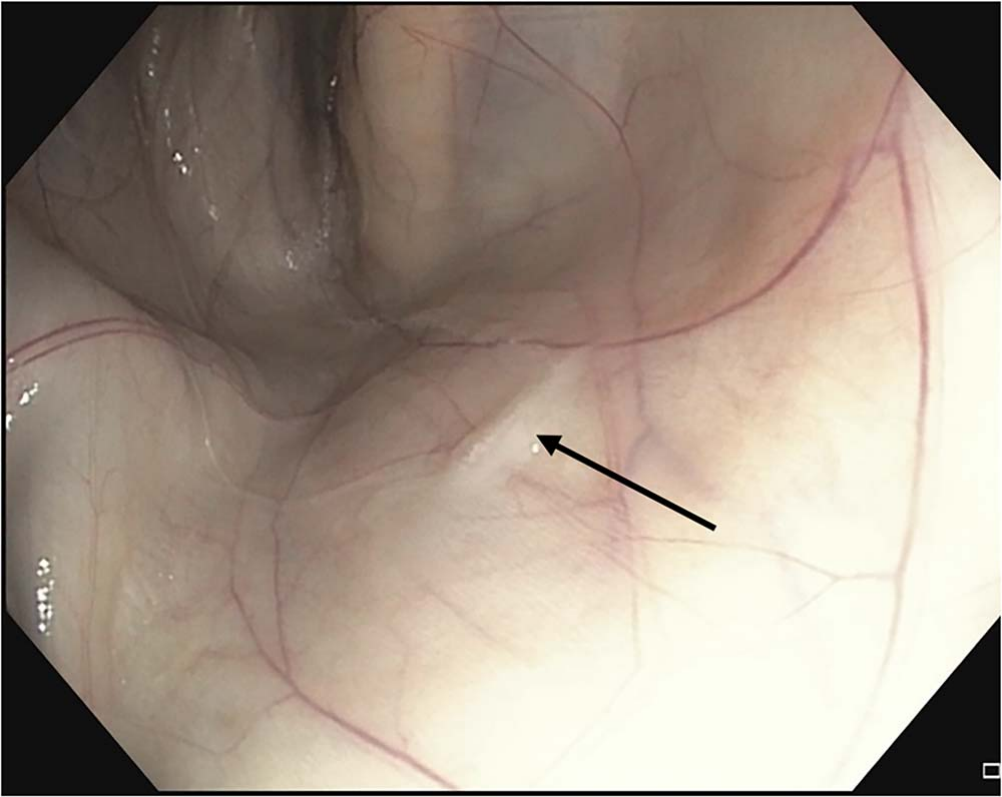
Mucoid secretions (black arrow) on the floor of the medial compartment of the left GP (GP inflammation score 2). Abbreviation: GP = guttural pouch.

**Figure 3:**
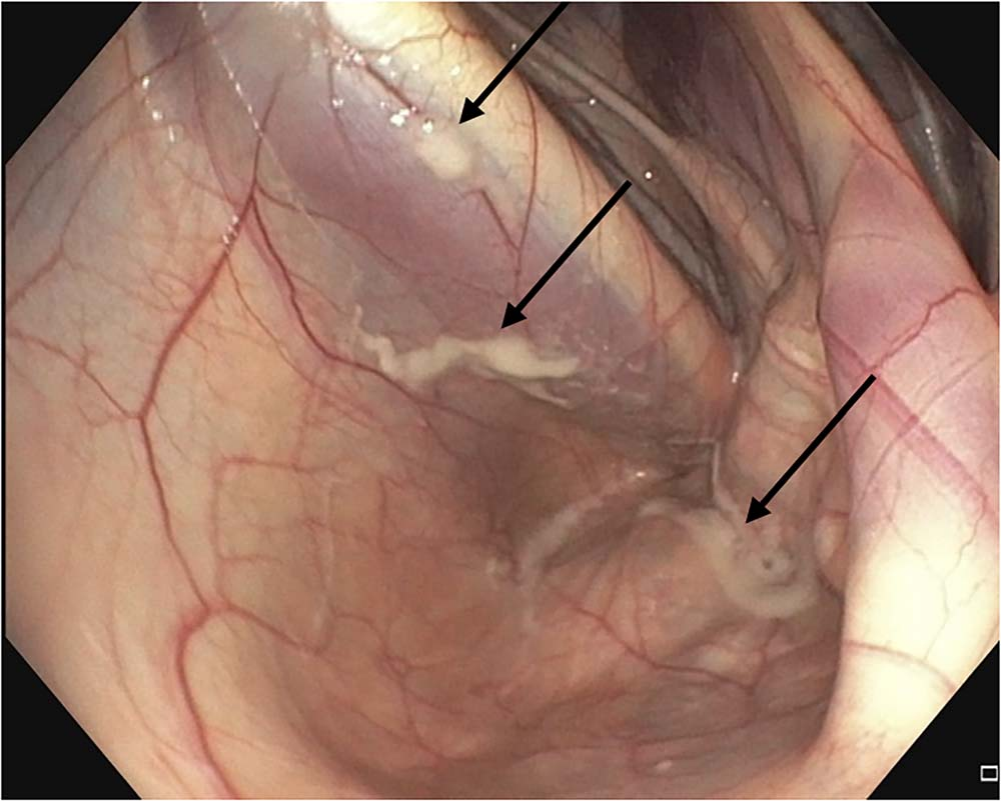
Purulent coalescent secretions (black arrows) on the floor and the medial wall of the medial compartment of the left GP (GP inflammation score 4). Abbreviation: GP = guttural pouch.

At least one abnormality was recorded in a GP in 65 horses (37.6%). Lymphoid hyperplasia was present in 40 horses (Figure 1), and 44 had secretions in at least 1 pouch (24 mucoid and 20 purulent material, Figures 2 and 3). Enlargement of retropharyngeal lymph nodes was recorded in 1 horse. No fungal plaque indicative of GP mycosis was observed.

Guttural pouch lymphoid hyperplasia was not associated with horse activity (racing vs. non-racing), and no correlation with age was identified (Spearman’s ρ = −0.157).

**Figure 4:**
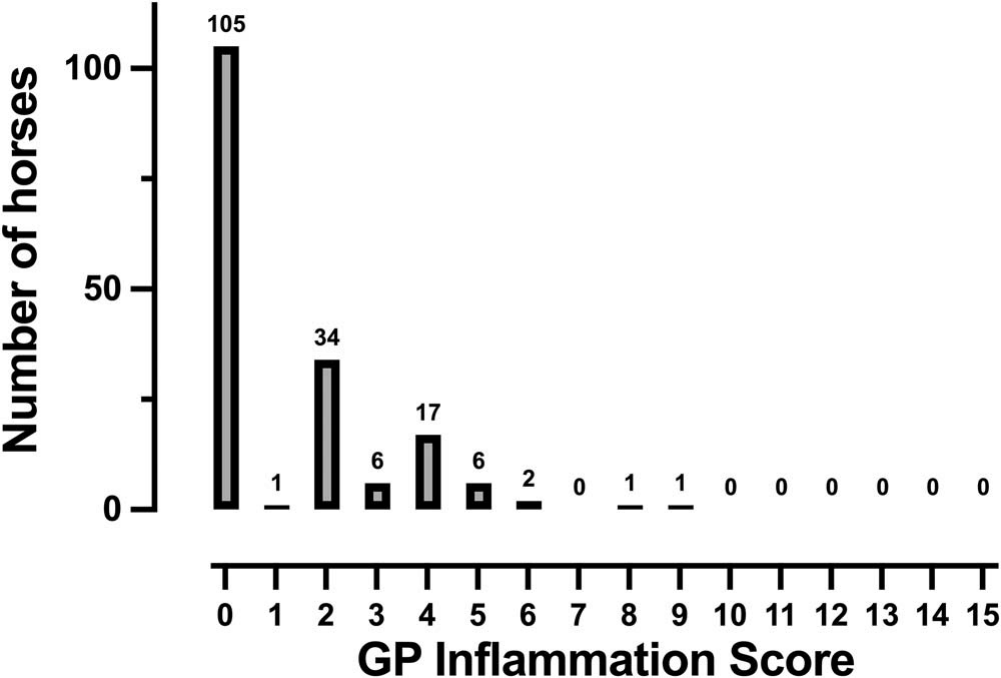
Distribution of the GP inflammation score in the study group. Abbreviation: GP = guttural pouch.

Guttural pouch inflammation score ranged from 0 to 9, with a median score of 1 (IQ1 0, IQ3 6; Figure 4).

No association was found between GP inflammation score and housing environment (stall vs pasture) or activity (racing vs non-racing), and no correlation was observed between GP inflammation score and age (Spearman’s ρ = −0.057).

#### Bacterial detection on guttural pouch

Bacteriology was performed on 7 samples when purulent secretions were observed on endoscopy. All yielded bacterial growth. *Streptococcus equi* subsp. *zooepidemicus* (>100 000 CFU/mL) was identified in 5 samples, *Pseudomonas* spp. in 2 samples (5000-25 000 CFU/mL), *Pantoea agglomerans* in 1 sample (>100 000 CFU/mL), *Psychrobacter phenylpirivicus* in 1 sample (>100 000 CFU/mL), and *Bordetella bronchiseptica* in 1 sample (>200 000 CFU/mL). Median GP inflammation score for horses with positive bacteriology was 3 (min 2, max 4).

Two horses were positive on real-time (RT)-PCR for *S equi* subsp. *equi* but not on bacterial culture. Guttural pouch inflammation score for these 2 horses was 0.

#### Fungal detection on guttural pouch

Positive microscopic detection of fungal elements was obtained in 1.2% (2/173) of samples on cytology (Figures S2 and S3). They were not phagocytosed.

Positive mycology was obtained in 6.4% (11/173) of samples. Four genera were identified, with the most commonly isolated genera being *Aspergillus* spp. (7/11) and *Penicillium* spp. (6/11) alone or in combination with other fungi.

#### Groups constitution

**Figure 5:**
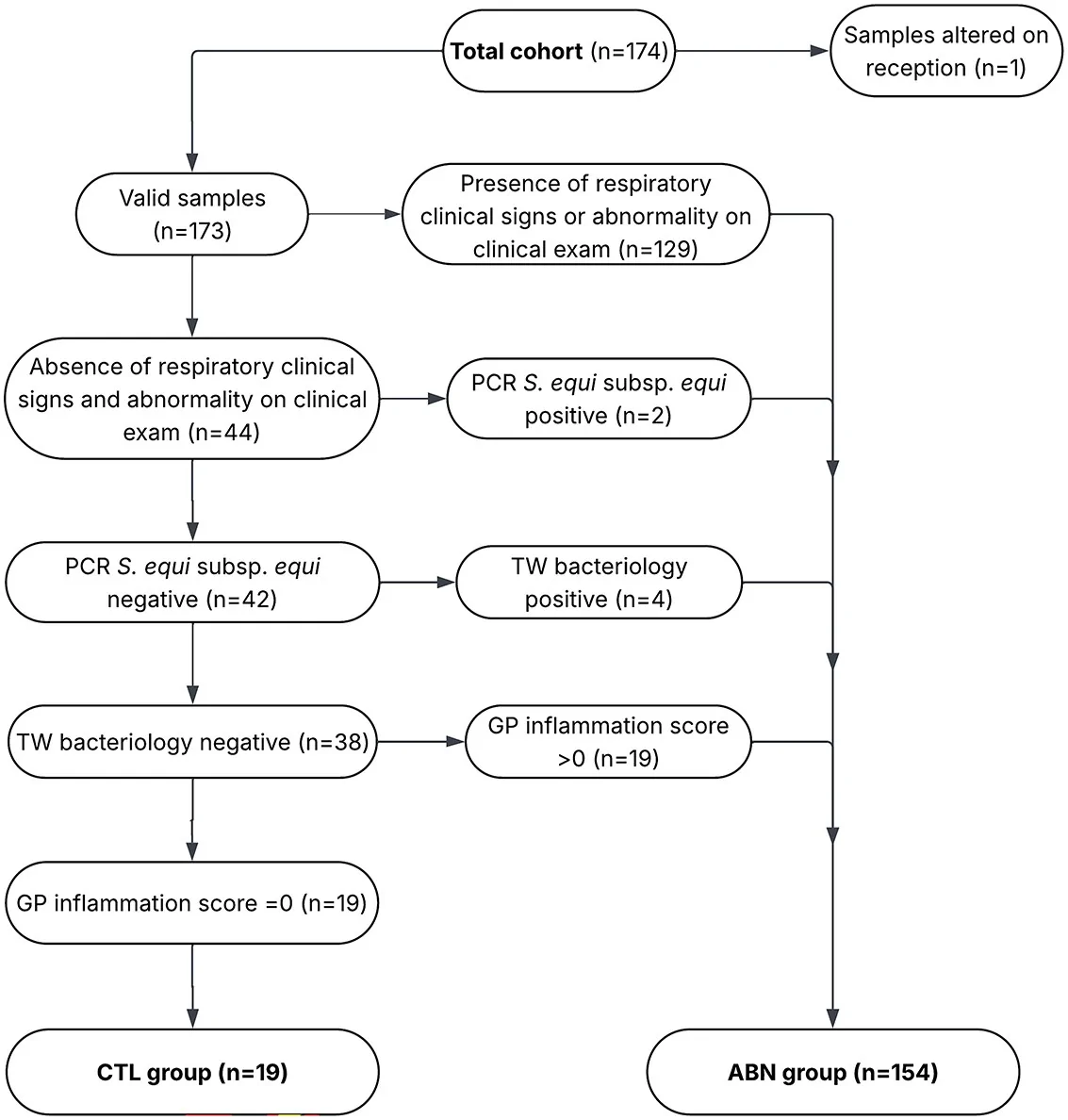
Flowchart of study cohort group constitution. Abbreviations: ABN, abnormal; CTL, control; GP, guttural pouch; TW, tracheal wash.

Among the 173 horses, 129 horses had abnormalities on respiratory examination, 2 horses were positive on PCR for *S equi* subsp. *equi*, 4 horses had a positive bacteriology on TW, and 19 horses had abnormalities on GP endoscopy. Overall, the control group contained 19 horses (Figure 5).

#### Cytology of guttural pouch

Median volume retrieved from GP was 32 mL (± 8 mL).

The control group (*n* = 19) was used to define a normal cytology. Median cell count/μL was 30 (IQ1 20, IQ3 55; min 10, max 90 cells/μL). Cytological examination revealed the presence of only 3 types of leukocytes: neutrophils, macrophages, and lymphocytes (Table 1; Figure S4), and a variable proportion of epithelial cells (Figure S5).

**Table 1:** Cytology of GP fluid in control horses (*n* = 19).

	**Cells/mm** ^**3**^	**Neutrophils (%)**	**Macrophages (%)**	**Lymphocytes (%)**
**Median**	30	0	0	0
**IQ1**	20	0	0	0
**IQ3**	55	0.2	0.2	0.1
**Minimum**	10	0	0	0
**Maximum**	90	0.5	0.5	0.2

Abbreviation: GP, guttural pouch.

Normal cytology was characterized by the absence of cells, or the presence of rare cells, which could be either neutrophils, macrophages, or lymphocytes.

Racehorses had significantly higher neutrophil proportions in GPs (median 0.2; IQ1 0, IQ3 75; min 0, max 95) compared to non-racing horses (median 0; IQ1 0, IQ3 0.2%; min 0, max 90; *P* < .001; Figure S6).

A moderate positive correlation was identified between neutrophil proportion in GP and GP inflammation score (Spearman’s ρ = 0.41).

Among the 154 horses in the abnormal group (Figure 5), neutrophil percentages were distributed as follows: 0%-0.99% (*n* = 101), 1%-20% (*n* = 3), 21%-40% (*n* = 3), 41%-60% (*n* = 5), 61%-80% (*n* = 20), and 81%-100% (*n* = 22).

**Figure 6:**
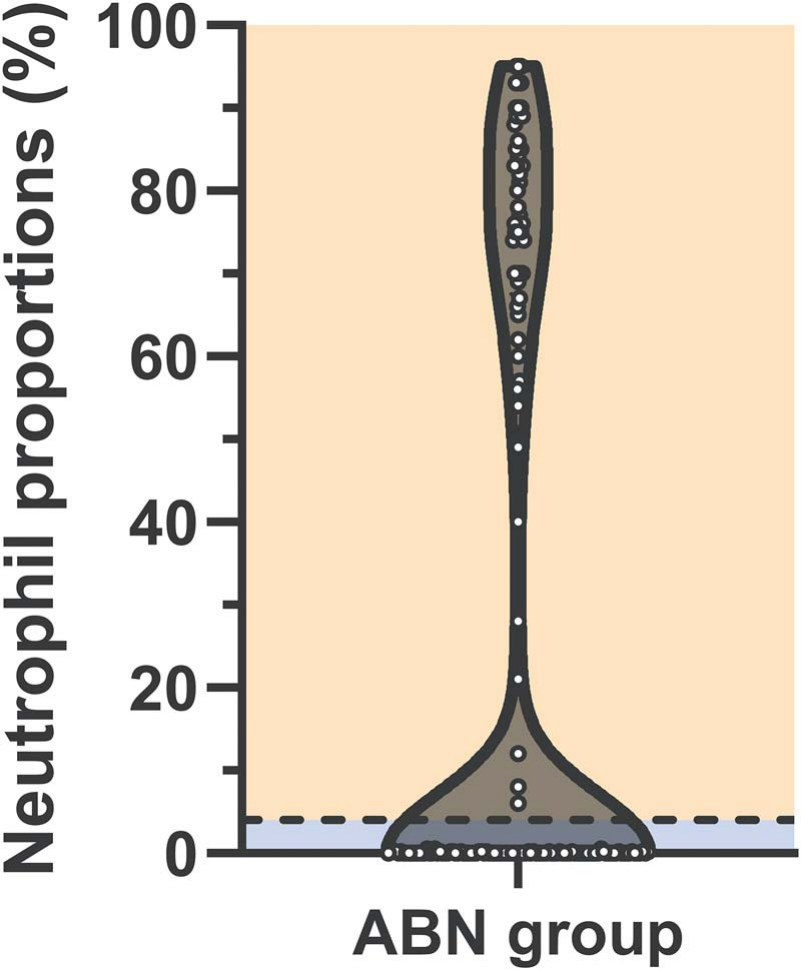
Repartition of the neutrophil proportions in GP fluid in abnormal horses. Dashed line is at 4%. In black, horse distribution with 1 dot per horse. The ABN1 group is located below the dotted line, whereas the ABN2 group is located above the dotted line. Abbreviations: ABN = abnormal; GP = guttural pouch.

Abnormal horses were further subdivided into 2 groups according to GP fluid neutrophil proportions: group ABN1 included 101 horses with no or rare neutrophils (<1%, median 30 cells/μL, range 5-360; Figure 6) and group ABN2 included 53 horses with neutrophils ≥ 1% (median 100 cells/μL, range 30-600; Figures S6 and S7).

#### Association between detection of infectious agents in GP, endoscopic findings, and cytology

When comparing horses with positive mycology on GP fluid to horses with a negative mycology, no difference in cell count or neutrophil proportion was observed.

For the 2 horses positive for *S equi* subsp. *equi* by PCR, both had low cell counts (40 and 180 cells/μL, 0.2% and 0% neutrophils, respectively).

For the 7 horses with positive bacteriology in GP, percentage of neutrophils was high in all horses (67%-93% neutrophils), but cell counts remained low (0-570 cells/μL).

#### Comparison between ABN1 and ABN2 groups and GP endoscopic images

Guttural pouch lymphoid hyperplasia was significantly more prevalent (*P* = .002) in ABN2 horses (22/53, 41.5%) than ABN1 (18/101, 17.8%). Guttural pouch secretions were significantly more prevalent (*P* < .001) in group ABN2 (26/53, 49.1%) than ABN1 (18/101, 17.8%).

Mucoid secretions were significantly less prevalent (*P* = .006) in ABN2 (8/26, 30.8%) than ABN1 (14/18, 77.8%).

Purulent secretions were significantly more prevalent (*P* = .015) in ABN2 (16/26, 61.5%) than ABN1 (4/18, 22.2%).

A significant association (*P* = .003) was observed between GP and PLH, and PLH (grade > 1/4) was significantly more prevalent (*P* < .001) in ABN2 (33/52, 64.2%) than ABN1 (32/97, 35.6%).

**Figure 7:**
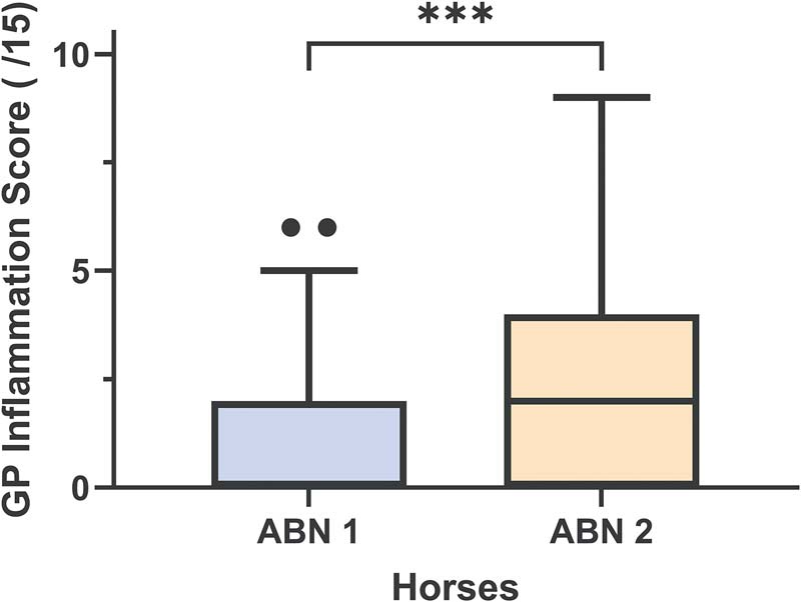
Association between GP inflammation score and ABN groups. The dots represent outliers. The 3 asterisk indicate a statistically significant difference between ABN1 and ABN2 with *P* < .001. Abbreviations: ABN = abnormal; GP = guttural pouch.

Horses in ABN2 had a significantly higher GP inflammation score than horses in ABN1 (*P* < .001; Figure 7).

#### Association between lymphoid hyperplasia in GP and PLH

Pharyngeal lymphoid hyperplasia grade was recorded in 97/101 horses in group ABN1 and 52/53 in group ABN2. Horses with PLH > 1 were 32/97 (35.6%) in group ABN1 and 33/52 (64.2%) in group ABN2. Pharyngeal lymphoid hyperplasia grade > 1 was significantly more prevalent in group ABN2 (*P* < .001).

A significant association was observed between GP lymphoid hyperplasia and PLH, and PLH (grade > 1/4) was significantly more prevalent in ABN2 (63.5%) than ABN1 (33%).

## Discussion

### Reference value of GP cytology in healthy horses

This study confirmed that GP fluid cytology of control horses (without respiratory disease) is typically characterized by the absence or a low number of cells, which can be neutrophils, macrophages, or lymphocytes. This low cellularity is reported in studies conducted on clinically healthy horses.^4–8^ Previously, GP inflammation was arbitrarily classified as follows: normal when neutrophils comprised less than 5%, mild to moderate inflammation when neutrophil proportion ranged from 5% to 25%, and marked inflammation when exceeding 25%.^4^ This classification was mainly based on reference values for cytological profiles from nasal fluid lavage,^13^ TW,^13–15^ and bronchoalveolar lavage fluid (BALF).^13^ However, in the present study, the proportion of neutrophils in control horses—confirmed as free of respiratory disease both clinically and analytically—was below 1%, suggesting that normal GP cytology is even less cellular than previously described.

Several factors can influence GP cytological findings, including the sampling method and the volume of instilled lavage fluid. Endoscopic-guided sampling allows direct visualization of the GP mucosa and enables targeted collection of secretions or exudate from specific anatomical areas, including the pharyngeal openings or medial and lateral compartments of the GP. This technique improves diagnostic accuracy, particularly in cases of localized disease such as empyema or fungal plaques. However, it requires specialized equipment and technical expertise, and might be less feasible in uncooperative or unsedated horses. In contrast, the transcutaneous approach, usually performed via ultrasound-guided or blind aspiration through the Viborg’s triangle, offers a less technically demanding alternative that can be performed without endoscopy. Nevertheless, it provides limited control over the sampling site, does not allow for visual assessment of the mucosa and carries a risk of inadvertent damage to adjacent vessels or nerves. Total cell counts might be influenced by sampling technique, with more frequent contact between the endoscope and the mucosa potentially brushing off epithelial cells and increasing overall cellularity.^8^ In our study, the highest observed total cell count was 600 cells/μL, which aligns with reported values, regardless of whether lavage was performed transendoscopically or percutaneously.^4^^,^^8^ Although this aspect was not formally investigated, we did not observe increased cellularity in horses that moved during sampling, as the majority of horses in this study were sedated to minimize movement and improve sampling consistency. Finally, in the current study, a lavage volume of 40 mL was instilled in each pouch, with a median recovery volume of 32 mL, representing 80% of the instilled amount. This recovery rate is consistent with previous findings.^4^^,^^8^

### Variables associated with increased neutrophil proportion in GP

An increase in the neutrophil proportion in the GP fluid might occur under various conditions. In one study, horses undergoing light exercise exhibited a higher proportion of neutrophils in GP samples compared to those subjected to heavy exercise.^4^ The authors suggested that more frequent opening of the pharyngeal orifices during intense exercise could facilitate drainage of secretions and clearance of contaminants that could trigger a neutrophilic response within the pouches. In our study, an association was found between the horses’ level of physical activity and GP cytology, with racing horses having higher neutrophil proportions in their GP compared with non-racing horses. Beyond exercise intensity, other factors such as age, training status, stable environment, transportation, and management conditions might also have contributed to the observed cytological differences in racehorses, and warrant further consideration in future studies.

Another study reported that maintaining an elevated head position for more than 12 h significantly increased both neutrophil proportion and overall cellularity in GP fluid.^6^ It is proposed that periods of lowered head posture could be required for normal clearance of secretions from the equine lower respiratory tract.^16^ In our cohort, evaluating this factor was challenging because transport time to the clinic was relatively brief (less than 2 h), and horses sampled at their stables (ie, without transport) were predominantly racehorses, which introduced a bias. Consequently, the relatively brief duration of transport for hospital-referred horses might not have been sufficient to alter GP cytology profiles.

Infectious agents in the GP can also influence cytological findings.^4^^,^^6^ A positive bacterial culture in the GP is associated with increased neutrophil counts in some studies,^4^^,^^6^ even when bacteria usually considered non-pathogenic were isolated.^4^ Conversely, one study found no association between a positive GP bacterial culture and elevated neutrophil count,^8^ although only bacteria of questionable pathogenicity were identified. Interpretation of such findings depends not only on the bacterial genus—distinguishing commensals from true pathogens—but also on the bacterial load. While a recent study described the bacterial and fungal microbiota present along the upper and lower respiratory tract of healthy horses,^17^ it involved a limited sample size, and neither the prevalence nor the quantity of bacteria in the GP was assessed. In the TW, a commonly accepted bacterial pathological threshold is 10^3^ CFU/mL.^18^ Previous studies on GP samples often did not report bacterial quantities. In the current study, all isolated bacterial loads exceeded 10^3^ CFU/mL, supporting their potential role in active infection and subsequent neutrophilic inflammation observed in the GP.

The 2 horses in our study that tested positive for *S equi* subsp. *equi* by PCR but not bacteriology showed cytological profiles comparable to those of control horses. This finding contrasts with earlier studies where *S equi* subsp. *equi* infection was linked to marked neutrophilic inflammation in the GP.^4^^,^^9^ This discrepancy could at least partly be due to differences in diagnostic methods: the earlier studies performed bacterial culture, whereas our study employed real-time PCR. While PCR is approximately 3 times more sensitive than culture, it does not distinguish between dead and live organisms.^19^ It is therefore possible that the 2 positive horses in our study did not have active infection.

Regarding fungal elements, although in the current study only a small proportion of horses (6.4%) showed positive fungal culture from GP samples, this was not associated with an increase in cellularity or neutrophil proportion. This low detection rate is consistent with previous reports.^6^^,^^7^ The genera identified (*Aspergillus* and *Penicillium*) were similar to those reported in other studies^7^^,^^17^ and could either represent simple inhalation or part of the normal fungal microbiota (mycobiota) of the GP.

### Correlation between endoscopic findings and GP cytology

The present study demonstrated a correlation between cytological evidence of inflammation and endoscopic signs suggestive of GP inflammation, such as lymphoid hyperplasia or the presence of purulent secretions. The combined use of endoscopy and GP cytology can also be helpful in monitoring the resolution of inflammation or infection in cases requiring follow-up, such as empyema or strangles infection.

The presence of secretions could indicate an underlying infection or a noninfectious inflammatory process, as is the case for equine asthma, which is characterized by an increased amount of tracheal mucus without identification of an infectious agent.^20^ In the current study, it was not possible to clearly distinguish between infectious and noninfectious causes of GP inflammation, as bacteriological analysis was not systematically performed for all cases. However, bacteriology was selectively performed—at the clinician’s discretion—in horses with abnormal endoscopic findings, typically the presence of purulent material. In all these cases, samples yielded positive bacteriology, with high bacterial loads and identification of pathogenic organisms, and degenerate neutrophils were observed on the smear, which is often indicative of a bacterial infection. These preliminary results confirm that the presence of purulent secretions in the GP is frequently associated with a neutrophilic inflammatory response and is often of bacterial origin.

Nonetheless, several horses displayed inflammatory cytology despite normal endoscopic findings, while others showed endoscopic evidence of inflammation with normal cytology. These inconsistencies highlight the need for further research to clarify the relationship between GP endoscopy and cytology. Additional diagnostic tools, such as advanced imaging or histopathology, might provide greater insight into the underlying disease.

In this study, GP lymphoid hyperplasia was significantly more prevalent in ABN2 horses—those exhibiting an inflammatory cytological profile in the GP. This finding supports the interpretation that, similar to PLH, lymphoid hyperplasia of the GP is a marker of local inflammation. Lymphoid hyperplasia of the GP was also associated with the presence of PLH in our study. Pharyngeal lymphoid hyperplasia is characterized by inflammation of local lymphoid tissue in the nasopharyngeal region,^21^ and is thought to result from exposure to various irritants, allergens, and viral or bacterial agents.^11^ In young horses up to 5 years old, PLH is a common, self-limiting condition.^11^ In the present study, the association between PLH and GP lymphoid hyperplasia suggests a possible extension of inflammation from the pharynx to adjacent GP structures. However, this relationship remains unclear; some horses with grade 4 PLH showed no evidence of GP lymphoid hyperplasia, while others with no PLH had marked GP involvement.

In a previous study,^12^ the authors proposed a scoring system for upper airway inflammation that combined an established grading system for PLH with a specific score for GP inflammation (based on lymphoid follicles, enlarged retropharyngeal lymph nodes, edema, or exudate within the pouch). Based on this grading system, we developed a GP inflammation score grounded on the same framework, but expanded through a comprehensive description of endoscopic findings. This score systematically evaluates the various visual signs of inflammation: mucosal hyperemia or hyperplasia, the character and amount of exudate, and lymph nodes enlargement. Each criterion is graded precisely to standardize interpretation and improve reproducibility between observers. Our results showed a moderate correlation between this endoscopic inflammation score and the percentage of neutrophils in the pouch, suggesting that the visual assessment partially reflects the intensity of cytological inflammation.

Upper airway inflammation scores decrease when horses are kept at pasture.^12^ In our study, GP hyperplasia was not associated with housing, supporting the hypothesis that exposure to dust and environmental allergens might not contribute to the development of GP lymphoid hyperplasia.

## Limitations

The main limitation of this study was the absence of systematic bacteriology of GP fluid. As this investigation formed part of a broader study not primarily focused on characterizing the GP microbiota, bacteriological testing was performed selectively at the clinician’s discretion and for clinical purposes only. Additional testing for infectious agents such as herpesvirus type 2 or 5 would also have been valuable, as these viruses are believed to contribute to PLH.^11^

The sensitivity of cytological evaluation could have been affected in cases where the lavage fluid appeared clear and contained only a few cells. However, in such cases, the entire slide was carefully examined to obtain an accurate cell count and minimize this potential source of bias.

Finally, even though both GPs were sampled, cytology was performed on pooled fluid. The GP inflammation score was not calculated for each GP, but was combined for both pouches. Further studies should consider performing cytology and scoring for each GP individually to better capture the heterogeneity of disease, improve the accuracy of the scoring system, and more precisely guide decision-making.

## Conclusion

In this study group, GP fluid cytology in control horses was characterized by low cellularity. We defined a normal GP cytology as having cell counts < 90/μL and neutrophils < 1%.

Our results highlight a correlation between GP cytology and endoscopic observations. Combining these 2 diagnostic approaches could enhance the accuracy of GP inflammation diagnosis. Given the widespread availability of endoscopy, it should be considered the first-line tool for GP screening. When abnormal findings are observed—such as purulent secretions or lymphoid hyperplasia—GP lavage and cytological analysis can confirm the presence of inflammation. Additional diagnostic tools, such as bacterial or fungal culture and PCR, should then be used to identify the underlying cause.

## Supplementary Material

Supplementary_material_aalag179

## Off-label antimicrobial declaration

The authors declare no off-label use of antimicrobials.

## Institutional animal care and use committee or other approval declaration

Approved by the regional Ethics Committee for Clinical and Epidemiological Veterinary Research (CERVO-2020-3-V). Owners gave consent for their animal’s inclusion in the study.

## Appendix

### Study group

Data concerning housing conditions (indoor/outdoor), bedding (straw, wood shavings), hay forage (dry/soaked/steamed), activity (training/resting), occurrence of poor performance or exercise intolerance, as well as reported respiratory clinical signs (cough, dyspnea, nasal discharge, noise during exercise, increased respiratory effort at rest, epistaxis), were collected. Horses that had received corticotherapy or antimicrobial medication within 2 weeks before examination were not included in the study.

Each horse was submitted to a thorough clinical examination by a European College of Equine Internal Medicine (ECEIM) diplomate or resident. If observed, the presence of cough, nasal discharge, nostril flaring at rest, or dyspnea was recorded. Rebreathing pulmonary auscultation was performed and abnormal pulmonary (wheezes/crackles) or tracheal sounds, induction of cough, and delayed recovery following removal of the rebreathing bag were recorded. Venous blood samples were collected for hematology and additional biochemical analysis when judged clinically necessary.

Horses that were not racing or competing within 10 days following sampling were sedated with detomidine (10 μg/kg IV, Sedomidine, Audevard), with the addition of butorphanol (10-20 μg/kg IV, Torphadine, Dechra) when needed. A nose twitch was used when necessary. For horses racing or competing within 4-10 days following investigation, the procedures were achieved using a nose twitch only, without sedation because of drug testing considerations.

### Guttural pouch endoscopy

A 120-cm-long videoendoscope (AOHUA, Medimage off-site; OLYMPUS in-house) was passed through one of the nostrils into the pharynx.

A metallic wire was used to raise the cartilage flap of the orifice of each guttural pouch, and the endoscope was then inserted into the pouch. A visual assessment was performed, and images were recorded. Both compartments (medial and lateral) of each pouch were visualized.

### Respiratory sampling

#### Guttural pouch lavage

A single-use double-lumen sterile catheter (Mila International Inc.) was passed through the canal of the endoscope, and 40 mL of warmed (37°C) sterile saline solution (0.9% NaCl, B. Braun) was infused and retrieved within each guttural pouch. The saline was sprayed throughout the entire GP, including both medial and lateral compartments, and was subsequently completely re-aspirated under endoscopic guidance, predominantly from the medial compartment, as its larger volume facilitates fluid recovery. The aspirated fluid of both pouches was pooled, and was then divided into a tube containing EDTA for cytological analysis, and a sterile dry tube for microbiology. Mycology and a polymerase chain reaction (PCR) for *S equi* subsp. *equi* were systemically performed on all samples. Bacteriological testing was carried out only on samples selected at the clinician’s request.

#### Tracheal wash (TW)

A 120-cm-long videoendoscope (AOHUA, Medimage off-site; OLYMPUS in-house) was passed through one of the nostrils into the pharynx. The endoscope was then inserted into the proximal trachea, and a single-use double-lumen sterile catheter (Mila International Inc.) was passed through the canal of the endoscope, and 40 mL of warmed (37°C) sterile saline solution (0.9% NaCl, B. Braun) was infused and retrieved. The aspirated fluid was placed into a sterile dry tube for bacteriological analysis.

All samples were kept chilled until handled by the laboratory, and all analyses were performed within 24 h of sampling.

#### Cytology

Direct smears and cytocentrifuged preparations were made from the pooled GP fluid. Cytocentrifugation was performed on 300 μL of pooled GP fluid (800 *g*, 10 min, Cytospin, Shandon). All slides were stained with May-Grünwald-Giemsa (MGG). A differential cell count was performed on at least 400 cells and the number of each cell type was recorded as a percentage of total nucleated cells, excluding epithelial cells.

#### Microbiology

Bacteriological examinations were performed as previously described.^10^ Additionally, real-time-PCR for *S equi* subsp. *equi* detection was performed.

### Statistical analysis

Statistical analyses were performed using GraphPad Prism 10.6.1 software. Data were presented as median and IQR unless stated otherwise. Univariable associations were assessed using a chi-square test with Yates’ correction for continuity. Comparisons and correlations were, respectively, performed using Mann–Whitney *U* test and Spearman’s rank correlation. Correlation was described as poor when ≤ 0.20, fair if 0.21 ≤ value ≤ 0.40, moderate if 0.41 ≤ value ≤ 0.60, substantial if 0.61 ≤ value ≤ 0.80 and good if value exceeds 0.80. Values of *P* < .05 were considered statistically significant.

## Abbreviations

GP: guttural pouch
MGG: May-Grünwald-Giemsa
PLH: pharyngeal lymphoid hyperplasia
