# Supplementary material for: Association between cytology and endoscopy of guttural pouches: a prospective study on 173 horses
Source: J Vet Intern Med. 2026 Aug 26;40(4):aalag179. doi: 10.1093/jvimsj/aalag179 (PMC13513758; doi:10.1093/jvimsj/aalag179)
Supplement: Supplementary file 1 [file supplementary_material_aalag179.zip › Supplementary_material__aalag179/Suppl_Caption.docx]

**Caption/legend for supplementary files**

Table S1 : Horses’ characteristics

Figure S1: Association between PLH grade and horses’ activity

The dots (points) represent outliers. The three asteriks indicate a statistically significant difference between racing and non-racing horses with p < 0.001

PLH: Pharyngeal Lymphoid Hyperplasia

Figure S2: Microscopic image of a fungal hyphal element in guttural pouch cytology, observed at 50× magnification.

Figure S3: Microscopic image of a fungal spore in guttural pouch cytology, observed at 50× magnification.

Figure S4: Microscopic image of a neutrophil in guttural pouch cytology, observed at 50× magnification.

Figure S5: Microscopic image of an epithelial cell cluster in guttural pouch cytology, observed at 10× magnification.

Figure S6: Association between GP neutrophil proportions and horses’ activity

The dots (points) represent outliers. The three asteriks indicate a statistically significant difference between racing and non-racing horses with p < 0.001.

Break in the y-axis to improve readability due to a change in scale.

GP : Guttural pouch

Figure S7: Microscopic image showing a high proportion of neutrophils in guttural pouch cytology, observed at 10× magnification.
