# Supplementary material for: Association between cytology and endoscopy of guttural pouches: a prospective study on 173 horses
Source: J Vet Intern Med. 2026 Aug 26;40(4):aalag179. doi: 10.1093/jvimsj/aalag179 (PMC13513758; doi:10.1093/jvimsj/aalag179)
Supplement: Supplementary file 1 [file supplementary_material_aalag179.zip › Supplementary_material__aalag179/Table_S1.docx]

**Supplementary material for review and publication**

|  |  |  |  | Study population |
| --- | --- | --- | --- | --- |
| **Discipline** | Racehorse | Breed | Standardbred | 95 |
|  | Sport horse | Total |  | 26 |
|  |  | Breed | Warmblood | 17 |
|  |  |  | Standardbred | 1 |
|  |  |  | Pony | 3 |
|  |  |  | Draft | 0 |
|  |  |  | Other | 5 |
|  | Leisure horse | Total |  | 53 |
|  |  | Breed | Warmblood | 22 |
|  |  |  | Standardbred | 1 |
|  |  |  | Pony | 9 |
|  |  |  | Draft | 4 |
|  |  |  | Other | 17 |
| **Median age** (in years) | | | | 6 |
| **Sex** | Entire males | | | 41 |
|  | Geldings | | | 67 |
|  | Females | | | 66 |
| **Housing** | Pasture | | | 67 |
|  | Box with shavings | | | 15 |
|  | Box with straw | | | 92 |

**Table S1: Horses’ characteristics**
